# Quantitative investigation of moisture migration during microwave drying of coal slime dough through simulation and tracer analysis

**DOI:** 10.1038/s41598-025-95646-y

**Published:** 2025-04-02

**Authors:** Fei Wang, Nan Tian, Lei Ren, Kai Zhang, Jing Wang, Yuanyuan Zhang

**Affiliations:** 1 Engineering Research Center of Ministry of Education for Resource Efficiency Enhancing and Carbon Emission Reduction in Yellow River Basin Shanxi University, Taiyuan, 030006 Shanxi China; 2 Beijing Key Laboratory of Emission Surveillance and Control for Thermal Power Generation, North China Electric Power University, Beijing, 102206 China; 3 State Key Laboratory of Clean Energy Utilization, Zhejiang University, Hangzhou, 310027 China; 4 No. 92 Wucheng Road, Taiyuan City, 030006 Shanxi Province China

**Keywords:** Coal slime, Microwave drying, Moisture, Migration, Energy science and technology, Environmental sciences, Thermodynamics, Phase transitions and critical phenomena

## Abstract

Moisture migration during the microwave drying process of coal slime is critical for improving drying efficiency and reducing energy consumption. Nevertheless, current methods for quantifying these migration behaviors remain insufficient. In this research, we employed a comprehensive approach that combines experimental investigations with multi-physical field simulations to quantitatively characterize the distribution and state of moisture within coal slime dough at different locations during microwave drying. The entire drying process was divided into three distinct stages based on temperature distribution: preheating, constant temperature, and reheating. During the preheating stage, as temperature rose, more than 74.1% of the initial water content within the central region of the slime dough underwent vaporization. creating a pressure gradient between the interior and exterior of the coal slime dough. In the subsequent constant temperature stage, over 45.5% of the remaining water content within the slime dough was driven to the surface by the pressure gradient, where it vaporized and diffused into the surrounding atmosphere. During the reheating stage, moisture was initially vaporized as steam and subsequently diffused into the atmosphere through inter-particle voids, due to the inherent difficulty in forming a continuous liquid bridge between particles at this stage. Furthermore, we examined the influence of particle size and dough diameter of coal slime on moisture migration and diffusion during microwave drying, thereby substantiating the diffusion mechanism. By integrating experimental and simulation data, this study provides a detailed understanding of the moisture migration mechanisms within coal slime dough during microwave drying. These findings are valuable for the design of efficient microwave drying technologies, particularly for drying materials with high-water content and viscosity, such as typical coal slimes and sewage sludge.

**Supplementary Information:**

The online version contains supplementary material available at 10.1038/s41598-025-95646-y.

## Introduction

Coal slime, a byproduct of raw coal washing, constitutes approximately 10% of the total mass of coal undergoing washing procedures, contributing to an annual production of 200 million tons in China^1,2^. Its high moisture content poses environmental issues and leads to resource waste when stored for extended periods, creating significant challenges for effective utilization. One promising solution is to use coal slime as a combustion material^3,4^ However, burning wet coal slime reduces its calorific value and lower the boiler combustion efficiency. Therefore, the efficient removal of moisture from coal slime, while minimization energy consumption, is crucial for optimizing its use.

Energy consumption during the drying process is primarily comprised of water heating (sensible heat), water vaporization (latent heat), and the energy required for moisture diffusion within the material^5^. Adjustment of the vapor partial pressure in the drying environment, as seen in vacuum drying, can reduce the boiling point of water, thus diminishing sensible heat requirements^6,7^. However, the potential for reducing drying energy consumption through such means is limited. Furthermore, the latent heat involved in the gasification process is fixed by water properties, highlighting the importance of decreasing the energy consumed for moisture diffusion and migration within the material during drying processes.

Numerous efforts have been devoted to reduce the energy consumption associated with moisture diffusion in traditional thermal-drying techniques. These strategies include reducing material thickness and increasing air velocity around the material^8^. For instance, studies on drying green microalgae paste have demonstrating that reducing paste thickness enhances water diffusion coefficients and increasing drying rates by exploring the drying characteristics, energy requirements, and kinetics of green microalgae paste^9^. Similarly, the drying energy consumption of cumin seeds was reduced by increasing the critical fluidization airspeed in a fluidized bed^10^. Reduction in leather thickness during convective drying also improved moisture diffusivity^11^. However, the efficacy of these measures in reducing drying energy consumption is limited due to the inherent heat transfer mechanism, which involves heat conduction from the surface to the interior of the material, while moisture is transferred from the interior to the exterior^12^. This mismatch between heat and mass transfer results in uneven surface heating, leading to the formation of a crust that impedes vapor diffusion and increases drying energy consumption.

Microwave technology has gained extensive application in the drying of various materials, including food, minerals, and carbon-containing fuels, owing to its unique selectivity and uniform energy distribution^13–16^. During the microwave drying process, water molecules interact with the alternating high-frequency electric field created by microwave radiation, causing the dipoles to rotate and vibrate, thereby producing heat and raising the internal temperature of the material^17,18^. This process simultaneously induces the transfer of heat and moisture from the interior to the exterior^19^, rendering microwaves particularly effective for drying materials with high viscosity and moisture-content, such as sewage sludge, coal slime, and lignite.

Early research has focused on the theoretical and technical applications of microwave drying^20^, including the successful pilot-scale microwave systems for drying municipal sewage sludge^21^. Studies on the drying characteristics of coal slime have revealed that particle size and microwave power are key factors influencing the moisture diffusion coefficient^22^. The microwave-induced pressure and vapor further assist in moisture removal from biomaterials^23^, as evidenced by the pumping phenomenon observed in lignite through digital recordings^24^. While studies have visually detected liquid films on the sample surface, attributing them to pressure gradients that drive water diffusion from the interior to the exterior^25^, a 3-D model coupling electromagnetics, heat, and mass transfer was developed to explore moisture distributions during intermittent microwave drying^26^. However, the phase state (liquid or gaseous) and the quantitative migration of moisture remain unexplored during microwave drying. This aspect is critical for optimizing the desiccation of high viscosity and moisture materials.

In this study, we utilized sodium chloride (NaCl) as a tracer to investigate the migration and transformation of moisture within coal slime dough during microwave drying. This approach is based on the principle that NaCl, being water-soluble, migrates along with the water within the coal slime during the drying process. As liquid water containing dissolved NaCl moves between different regions of the sample, the mass ratio of NaCl to the dry matter remains constant because NaCl does not vaporize or decompose during heating. The evaporation of moisture from the slime dough results in an increased mass ratio between NaCl and dry matter. Leveraging this principle, we analyzed moisture content and chloride ion concentration at various drying stages and locations within spherical coal slime dough. This analysis allowed us to study the moisture migration and diffusion mechanisms throughout the entire microwave drying process. Additionally, we also employed multi-physics simulations to support our experimental findings. Due to the variations in coal washing and separation processes, the coal slurry produced in industrial production has different particle sizes. To further investigate the effect of particle size on the moisture diffusion mechanism during microwave drying, we evaluated the impact of the particle size and pellet diameter on the drying process by calculating the effective diffusion coefficient. Our research serves as a significant contribution to the understanding of microwave drying processes for spherical materials and provides insights for the enhancement of microwave drying technology designed for high-water content and high-viscosity granular materials.

## Experimental

### Materials

The coal slime utilized in this study was sourced from the Pingshuo Coal Gangue Power Plant located in Shanxi. Prior to experimentation, a comprehensive industrial analysis and elemental analysis were performed, with results summarized in Table 1. We can see that the slime has a larger moisture content and higher calorific value. The coal slime underwent a series of preparatory steps to facilitate subsequent experimentation. Initially, the raw coal slime was subjected to a drying process in an oven at 105 °C to remove excess moisture, until its mass no longer changed, ensuring the moisture content reached 0%. Subsequently, the dried coal slime was sieved to obtain coal slime particles with distinct size fractions, specifically falling within the size ranges of 0.106–0.125 mm, 0.125–0.15 mm, and 0.15–0.18 mm.

**Table 1:** Proximate and ultimate analysis of coal slime.

Sample	Proximate analysis (%)				Q_net, ad_ (J/g)	Ultimate analysis (%)				
	M_ar_	A_d_	V_d_	FC_d_^*^		*N*_d_	C_d_	H_d_	S_d_	O
Coal slime	25.08	29.32	26.97	43.71	16,891	0.94	48.99	3.25	1.34	16.16

Mar, Ad, Vd, FCd*, Q_net,d_ represent moisture on received basis, ash on dry basis, volatile matter and fixed carbon,and calorific value, respectively

### Experimental equipment

#### Microwave drying system

**Fig. 1:**
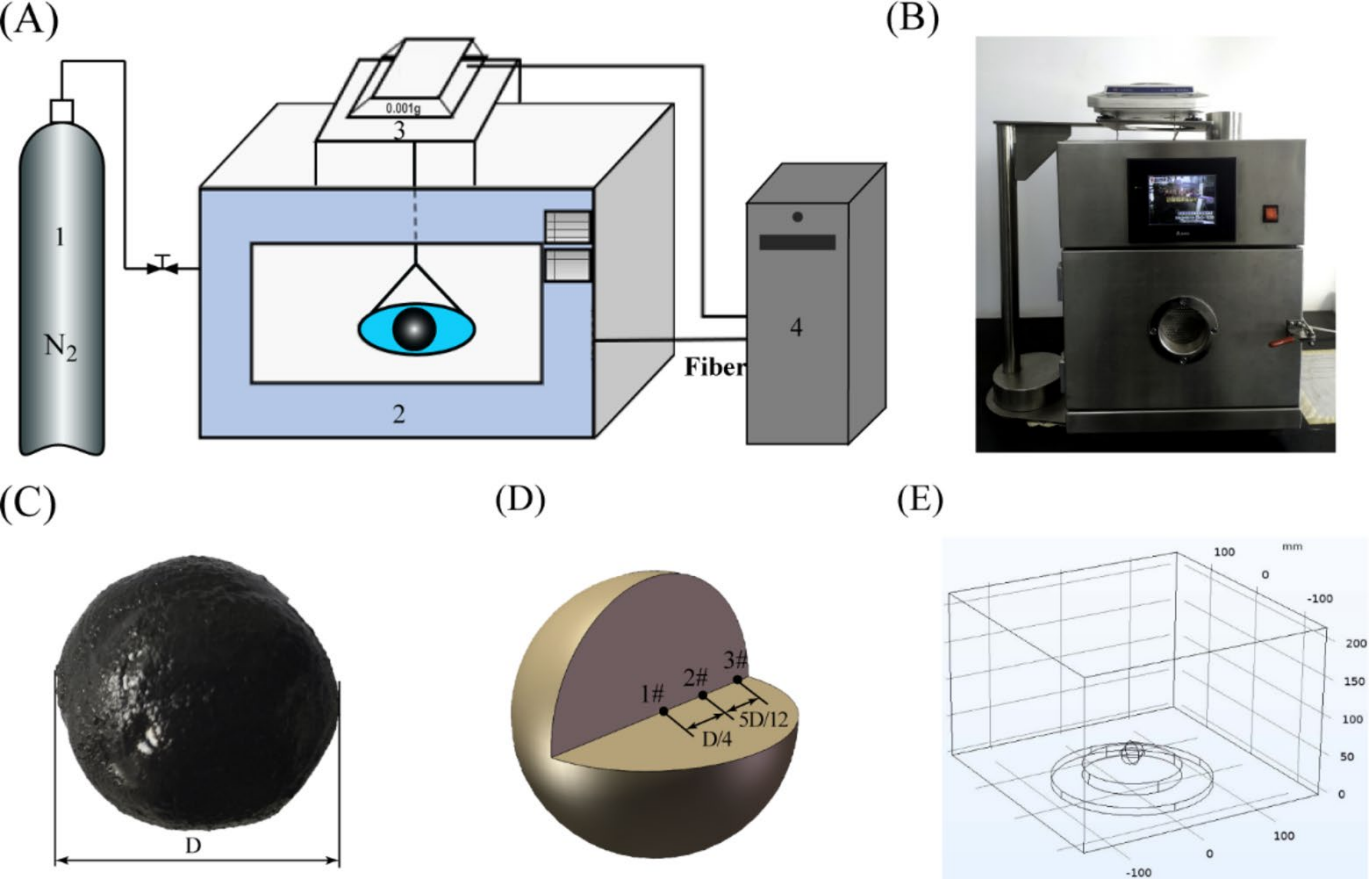
Schematic of the microwave drying system (**A**), including 1- Nitrogen gas cylinder. 2- Microwave device. 3- Digital balance. 4- Record control system. Device photograph (**B**). Photograph (**C**). Schematic diagram of coal slime (**D**) and microwave cavity geometry model (**E**). 1#, 2# and 3# are sampling and temperature measurement points.

The experiment utilized a customized intelligent microwave workstation, boasting a rated power of 1200 W and an operating frequency of 2450 ± 10 MHz. This microwave workstation featured an integrated mass (Mass sensor, LD510-2, Shenyang Longteng Electronics Corporation, Liaoning, China ) and temperature (Temperature sensor, GX01, Nanjing ORUN MICROWAVE Technology Corporation, Nanjing, China) measurement system, enabling real-time monitoring of coal slime temperature and weight changes. The weight measurement system demonstrated an accuracy of 0.01 g, while the temperature measurement system maintained a precision of 0.1 °C. Additionally, a gas system was integrated to replace evaporated vapor from the sample with nitrogen gas, thereby ensuring a consistent partial pressure of vapor on the surface of the slime dough. The schematic illustration of the microwave drying system, along with a photograph of the equipment, is presented in Fig. 1A and B.

#### Microwave drying process

(1) Preparation of wet slime samples: A 10% (wt%) sodium chloride solution was prepared. Subsequently, the sieved coal slime particles were uniformly mixed with the NaCl solution, ensuring thorough homogenization through stirring (The mass ratio of slime particles to sodium chloride solution is 1:0.335). After standing for 24 h, the water and slime particles were mixed to obtain slime with the same moisture content (25.08%) as the slime taken. (2) Coal slime dough formation: Coal slime dough with different diameters was created using spherical molds (as depicted in Fig. 1C). (3) Microwave drying setup: The coal slime dough was positioned at the center of the inner tray within the microwave device to maximize radiation intensity. The tray’s position remained fixed throughout the experiments, ensuring the reproducibility of each measurement. (4) Microwave drying operation: The microwave workstation was activated, facilitating the real-time recording of both the mass and temperature changes in the slime sample during the drying process.

### Analysis and characterizations

#### Moisture migration and transformation test method

To investigate the migration and transformation behaviors of moisture within wet coal slime dough, sodium chloride (NaCl) was employed as a tracer. The solubility of NaCl is less affected by temperature change. Therefore, NaCl served as a reliable indicator for studying moisture migration. Equal volumes of samples were extracted from three distinct points-1#, 2#, and 3#-within the coal slime dough, representing the central region, middle region, and surface, respectively (as depicted in Figure. 1(D) and the diameter D of the slime dough is 30 mm). Moisture content and NaCl concentration in these samples were analyzed to discern whether water from these points migrated to other areas or evaporated and subsequently diffused into the environment in the form of steam.

#### Measurement methods

Moisture content: The moisture content (wt%) of the coal slime was determined in accordance with the China National Standard for Determination of Total Moisture in Coal (GB/T211-2017). The moisture content reported in this study refers to the ratio of water mass to dry base mass. Temperature measurement: The temperature at different points (1#, 2#, and 3#) within the coal slime was measured using an infrared and optical fiber temperature measurement system. Chloride ion concentration: The concentration of chloride ions was measured following the China National Standard for Determination of Chloride (GB11896-89). Pressure measurement: The pressure inside the coal slime dough was measured using a self-made U-tube type manometer.

#### Effective diffusion coefficients

The effective diffusion coefficient (*D*_*eff*_) is a significant parameter reflecting the ability of water to diffuse and be removed. It can be calculated by Eq. (1).

1 $$\ln MR=\ln \left( {\frac{6}{{{\pi ^2}}}} \right)-\frac{{4{\pi ^2}{{\text{D}}_{\text{e}}}t}}{{{{\text{D}}^2}}}$$

where, D_e_ is the effective diffusion coefficient (m^2^/s); D is the diameter of the coal slime dough (m); *t* is heating time (s); *MR* is the ratio of the moisture content of coal slime at a certain time to the initial moisture content, $MR{\text{=}}\frac{{{M_t} - {M_e}}}{{{M_0} - {M_e}}}$; *M*_*t*_ is dry basis moisture content of the sample at time t; M_0_ is initial dry basis water content; M_e_ is the moisture content absorbed by the sample in the drying experiment which can be defined as 0 in the microwave drying experiment.

### Multi-physical field simulation

The multi-physical field simulation was conducted using the COMSOL software, aiming to model temperature changes, pressure distribution, and water vapor distribution during microwave drying of wet coal slime dough. The simulation employed a geometric model scaled at 1:1, mirroring the dimensions of the experimental device, as shown in Fig. 1 E. The computational domain was meshed with a free subdivision tetrahedral mesh, with distinct mesh size parameters applied to the air domain and the slime sample area to ensure result reliability, The mesh division of the geometric model was shown in Figure S1.

To accurately capture the heat and mass transfer mechanisms during the microwave drying process, the simulation also modeled the dynamic behavior of water migration and diffusion within the wet coal slime. Under the influence of microwave energy, the water molecules in the wet coal slime absorb electromagnetic energy, causing molecular vibrations that generate frictional heat, leading to a rapid increase in the internal temperature of the coal slime. The primary pathway for heat transfer is the direct interaction of microwave energy with the internal water, causing localized temperature spikes and inducing rapid evaporation of water. This evaporation is not limited to the surface but occurs simultaneously within the coal slime, resulting in the formation of significant temperature gradients. As the temperature rises, water migration and diffusion within the porous structure also take place. The movement of water vapor is primarily driven by temperature gradients and internal steam pressure gradients. The temperature gradient drives the migration of water from higher-temperature regions to lower-temperature regions, while the internal pressure gradient promotes the diffusion of steam from high-pressure to low-pressure areas. During this process, water migration involves not only the diffusion of liquid water but also the diffusion of vaporized steam following water evaporation.

Additionally, the model accounts for the phase transition between liquid and vapor states of water. As the water inside the coal slime is heated, liquid water gradually vaporizes and migrates toward the surface of the coal slime. Part of the water escapes directly through the surface, while another portion diffuses outward through the porous structure.

Several assumptions were made during the calculation process: (1) Parameters related to coal slime properties (e.g., electrical conductivity, electromagnetic wave loss rate, thermal conductivity, heat capacity, density, porosity, permeability, and initial water distribution) were considered uniform. (2) The boundary conditions for electromagnetic waves involved ideal conductors, while the temperature boundary conditions for the slime were based on natural convection and natural radiation heat dissipation at an external environment temperature of 20 ℃. (3) The boundary condition for Darcy’s law assumed a zero pressure at the outlet boundary of the slime surface.

The simulation encompassed the following steps: Electromagnetic Wave Frequency Domain Simulation: Calculated electromagnetic wave heat loss in the coal slime dough based on the Maxwell conservation Eqs^27,28^. The electric field vector *E* in the waveguide and microwave oven was solved under the specified rectangular port radiation. The equation was:

2 $$\nabla \times (\mu _{r}^{{ - 1}}\nabla \times E) - k_{0}^{2}({\varepsilon _r} - \frac{{j\sigma }}{{\omega {\varepsilon _0}}})E=0$$

where E is the electric field intensity (V/m), µr is relative permeability, σ indicates electrical conductivity (S/m), ωrepresents angular frequency (rad/s), and k_0_ is the wave number of free space.

After solving the electromagnetic wave heat loss power density, the heat loss power was coupled with the transient temperature field to simulate the endothermic phase change of liquid water in coal slime after heating under this power. Temperature Conservation Equation: Coupled electromagnetic wave heat loss power density with the transient temperature field to simulate the endothermic phase change of liquid water within the coal slime after heating. The overall temperature conservation equation was^29,30^:

3 $$\begin{gathered} \rho {C_p}u \cdot \nabla T+\nabla \cdot q=Q \hfill \\ q= - k\nabla T \hfill \\ \end{gathered}$$

where ρ is the density of the coal slime (kg/m^3^), Cp is the specific heat capacity (J/(kg/K)), u is the velocity field (m/s), T is the temperature (K), q is the heat flux density (W/m2), k is the thermal conductivity (Wm^−1^K^−1^), and Q is the internal heat source (W/m^3^).

Phase Change Mass Source: Utilized as the mass source term for steam inside the slime, factoring in phase change evaporation. Pressure Distribution: Employed Darcy’s law to compute the pressure distribution within the slime porous medium^31^. The equation was:

4 $$\begin{array}{*{20}{c}} {0=\nabla \cdot \left[ { - {{\text{p}}^2}+{\text{K}}} \right] - \left( {\mu {\kappa ^{ - 1}}+\beta \rho \left| {{{\text{u}}^{\text{2}}}} \right|+\frac{{{Q_{\text{m}}}}}{{\varepsilon _{{\text{p}}}^{2}}}} \right){\text{u}}+{\text{F}}+\rho {\text{g}}} \\ {\rho \nabla \cdot {{\text{u}}^{\text{2}}}={Q_{\text{m}}}} \end{array}$$

Which *Q*_*m*_ is the mass source term of the steam phase change (kg/(m^3^·s), p is the pressure (Pa), K is the permeability tensor (m^2^), µ is the dynamic viscosity (Pa*s), κ is the relative permeability (dimensionless), β is the inertial resistance factor (dimensionless), ρ is the density (kg/m^3^), u is the velocity field (m/s), ε_p_ is the porosity (dimensionless), F is the body force (N/m^3^), and g is the gravitational acceleration (m/s^2^). The parameters used in the simulation were shown in table S1 and table S2. This comprehensive approach allowed for a detailed analysis of temperature, pressure, and moisture distribution within the coal slime dough during microwave drying, providing valuable insights into the underlying physical processes.

## Results and discussion

### Microwave drying process and characteristics of coal slime dough

**Fig. 2:**
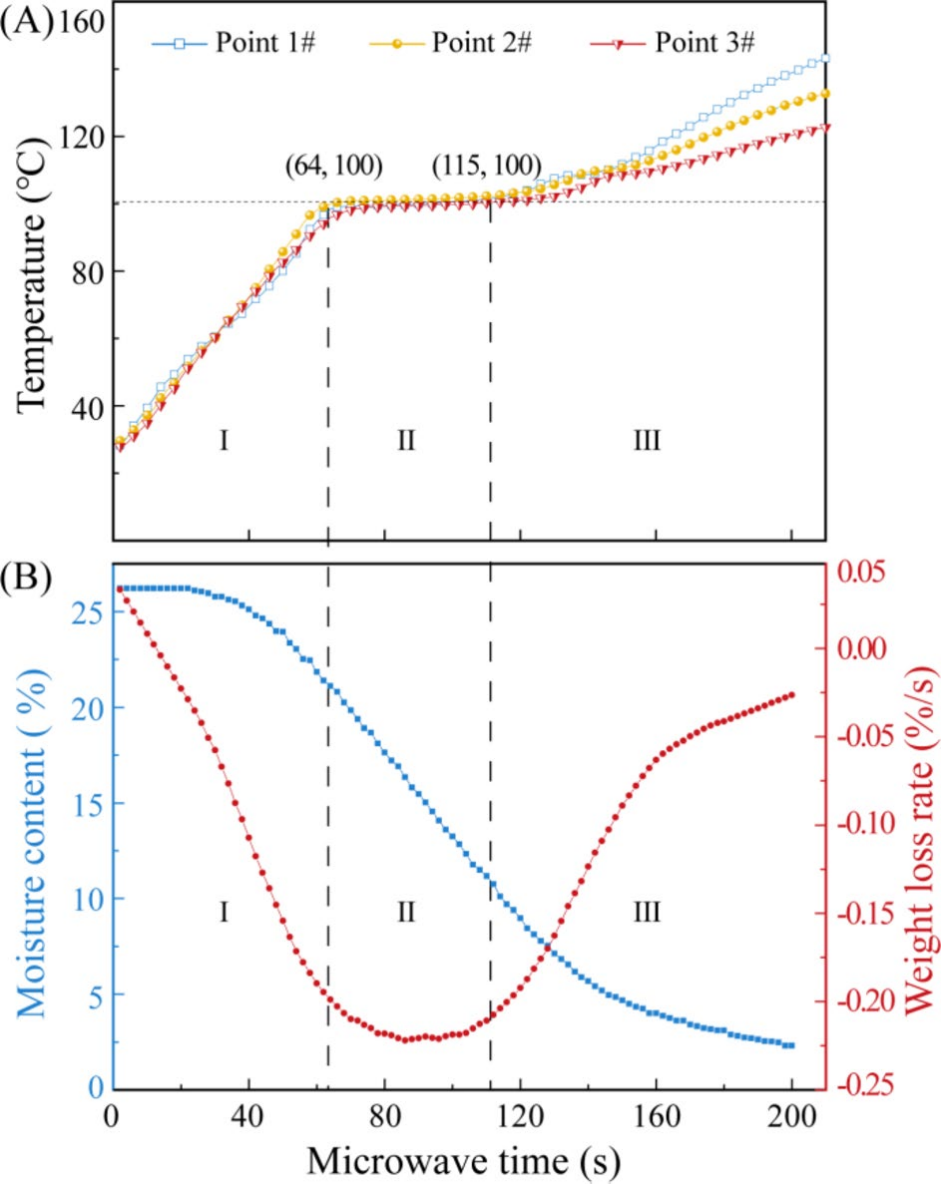
The temperatures at different locations and moisture content (g water/g dry base ) of coal slime dough, microwave power is 300 W, the interval is 5 s, the particle size of slime is 0.15–0.18 mm.

Sample temperature is a crucial parameter that significantly influences the drying process. Figure 2illustrates the temperature profiles at various locations within coal slime dough with a diameter of 30 mm, along with the corresponding moisture content. The temperature at all three monitoring points within the slime dough exhibited a gradual increase, starting from an initial temperature of 25 °C and rising to approximately 100 °C. Subsequently, the temperature continued to rise continuously after a period of stabilization at 100 °C. In previous research studies, the drying process was often divided into three stages based on the drying rate^32^. However, this approach lacked a clear feature point for stage demarcation. In this study, we employed the turning point in the temperature profile of the coal slime dough as a criterion to categorize the drying process into three distinct stages. This alternative approach enhances the precision of process characterization and helps in better understanding the dynamic changes occurring during microwave drying.

Stage I-Preheating Stage: The initial phase of the microwave drying process, termed “Stage I” or the preheating stage, was characterized by a rapid temperature increase. Within a span of 64 s, the temperature escalated from the starting point of 25℃ to 100℃. This remarkable temperature rise was attributed to the substantial input of microwave energy. During this stage, the amount of heat energy generated by microwave radiation surpassed the energy required for temperature elevation and moisture evaporation. Consequently, the weight loss rate of the coal slime dough exhibited gradual increments, leading to a reduction in moisture content by approximately 5%. This decline was primarily due to the evaporation of surface moisture from the dough.

Stage II-Constant Temperature Stage: “Stage II,” known as the constant temperature stage, extended from 64 s to 115 s. During this phase, the temperature of the coal slime dough remained relatively stable, hovering around 100 ℃. This temperature constancy resulted from an equilibrium state wherein the heat generated by microwave radiation and the energy essential for moisture evaporation within the slime dough reached a dynamic balance. Notably, the primary site for water evaporation during this stage was the surface of the coal slime dough exposed to the surrounding atmospheric environment. Due to the energy consumed by evaporation, the surface temperature was slightly lower than the nominal 100 ℃. This stage was marked by the highest rate of weight loss in the coal slime dough, corresponding to a substantial reduction in water content, which decreased by approximately 11%.

Stage III-Reheating Stage: In “Stage III,” referred to as the reheating stage, the energy absorbed by the wet coal slime dough from the microwaves exceeded the energy consumed by the remaining water’s evaporation. Consequently, the temperature of the slime dough continued to rise beyond the 100℃ threshold, commencing after the 115-second mark. Concurrently, the rate of moisture evaporation from within the slime decreased, leading to a further decline in its moisture content. This stage was characterized by ongoing temperature elevation and diminishing moisture levels within the coal slime dough.

### Migration and transformation mechanism of moisture

#### Distribution of moisture and concentration of chloride ion in different locations during drying process

**Fig. 3:**
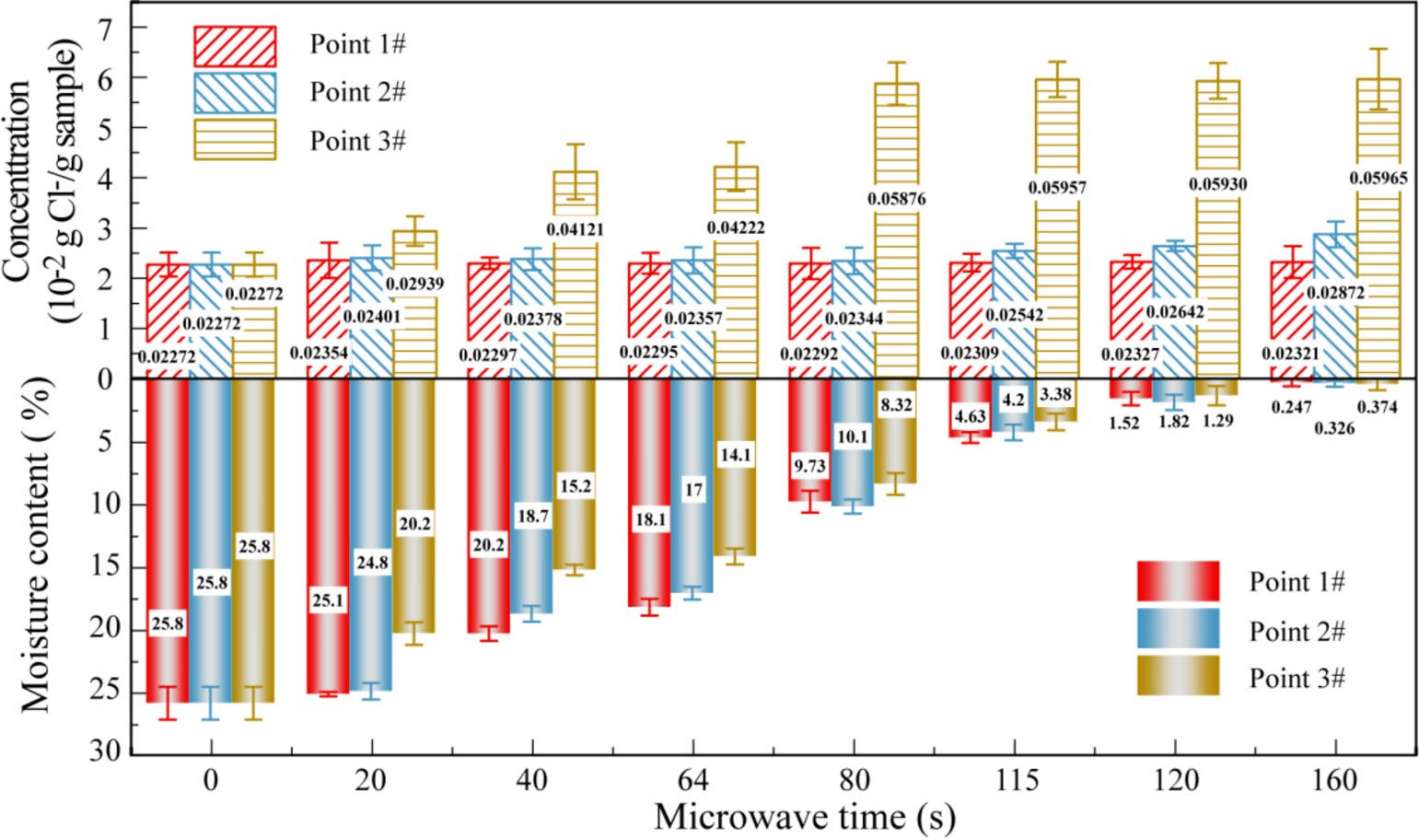
The moisture content and chloride ion concentration in different locations of coal slime dough with a diameter of 30 mm. The microwave power was 300 W, and the particle size of slime particles was 0.15–0.18 mm.

While the alterations in moisture content and temperature throughout the afore mentioned drying process provide insights into the overall water loss from the coal slime dough, they do not provide a detailed understanding of whether the reduction in moisture within the wet coal slime primarily occurred through liquid water migration or vapor diffusion. To address this critical question, we conducted a comprehensive investigation into the changes in moisture content and chloride ion concentration at various locations within the coal slime dough as a function of microwave irradiation time. This inquiry was undertaken to elucidate the intricate mechanisms governing moisture migration and transformation during microwave drying. The findings of this investigation are presented in Fig. 3.

The moisture content of samples from three designated points, 1#, 2#, and 3#, decreased as the microwave irradiation time extended. Conversely, chloride ion concentrations exhibited an upward trend. During the preheating stage (0–64 s), the moisture content at these points decreased by 7.7%, 8.8%, and 11.7%, respectively, while chloride ion concentrations increased by 0.023%, 0.085%, and 1.95%, respectively. Points 1# and 2# showed nearly unchanged chloride ion concentrations, suggesting that water from these points primarily migrated and diffused to the coal slime dough’s surface as liquid water during the preheating stage. Only a small portion of moisture evaporated into steam, increasing the partial vapor pressure inside the coal slime dough and promoting the movement of liquid water to its surface. Meanwhile, a significant increase in chloride concentration at point 3# indicated that water evaporation occurred on the dough’s surface.

In the constant temperature stage (64–115 s), moisture content at points 1#, 2#, and 3# continued to decrease to 4.6%, 4.2%, and 3.4%, respectively, with corresponding rising chloride ion concentrations. The increase in chloride concentration around points 1# and 2# was minor, suggesting that a small amount of water evaporated and diffused to the coal slime dough’s surface as steam during this stage. At point 3#, chloride ion concentration increased by 1.7%, indicating that, during the constant temperature stage, moisture in the wet slime dough continued to diffuse from the interior to the surface in the form of liquid water and eventually evaporated on the surface.

In the reheating stage (after 115 s), moisture content at points 1#, 2#, and 3# in the wet coal slime dough further decreased to approximately 0.2%. Significant increases in chloride ion concentrations were observed at points 1# and 2#. This occurred because the moisture content within the coal slime dough had decreased to a level where it was challenging to form a continuous liquid bridge between the slime particles, hindering liquid water diffusion to the surface via capillary action^24,33^. Consequently, liquid water from points 1# and 2# primarily evaporated within the coal slime dough and diffused to the surface as water vapor. Meanwhile, chloride ion concentration at point 3# remained stable at around 6%. This was attributed to the migration of the evaporation site from the initial surface to the inner surface of the coal slime dough during the microwave drying process, resulting in no further water evaporation at the area in contact with the environment.

#### Quantitative analysis of liquid water and gaseous water migration

In order to provide a quantitative description of water diffusion characteristics within various regions of the coal slime dough during microwave drying, we employed a calculus-based approach. For this purpose, we divided the coal slime dough with a diameter of 30 mm into three distinct regions, as depicted in Figure S2. Region 1: This region comprised a sphere with a radius of 5 mm. Region 2: It consisted of a spherical shell with an inner diameter of 5 mm and an outer diameter of 10 mm. Region 3: Similarly, this region was a spherical shell with an inner diameter of 10 mm and an outer diameter of 15 mm.

**Fig. 4:**
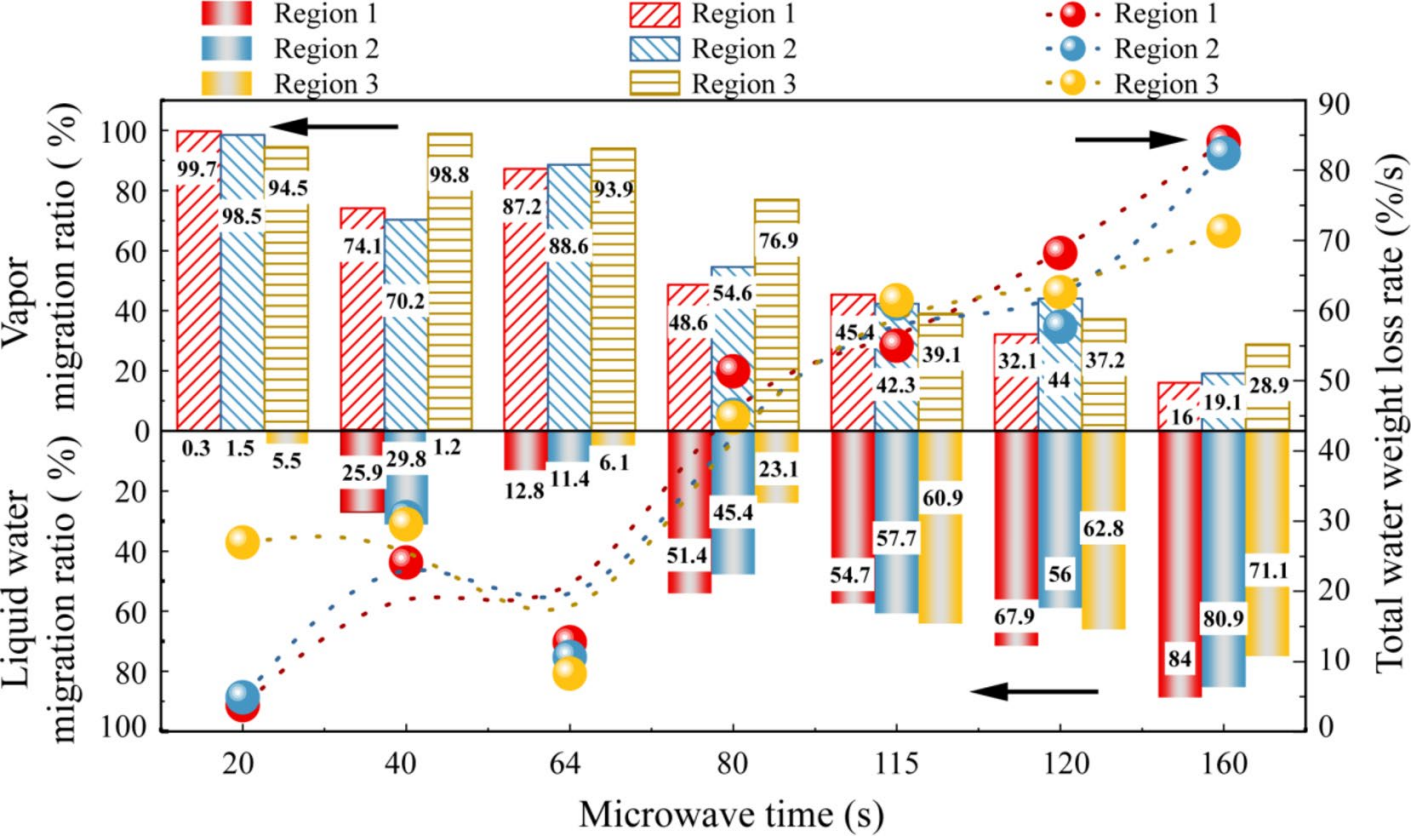
The total water weight loss rate of coal slime dough with a diameter of 30 mm and the migration ratio of liquid water and vapor in different regions.

To conduct a preliminary analysis of the total water weight loss rate and the migration ratio of liquid water and vapor within different regions at various times during the drying process, we utilized the water content and chloride ion concentration data obtained from the previous tests. These data served as the average values for the samples and were instrumental in quantifying the dynamics of water migration, as illustrated in Fig. 4.

As depicted in Fig. 4, the total water weight loss rate of the coal slime dough progressively increased with prolonged drying time. During the preheating stage, it was evident that the overall water loss rate in region 3 surpassed that in region 2 and region 1. This observation indicates that the initial stage of the drying process primarily witnessed evaporation occurring on the surface of the coal slime dough. Notably, at 64 s of microwave radiation time, a substantial 94.9% of the water weight loss in region 1 transformed into steam. This finding suggests that during the preheating stage, as the temperature increased, water within the central region of the slime dough primarily underwent gasification. These vaporized water molecules played a pivotal role in creating a pressure differential between the central region and the external environment, with the resulting pressure gradient serving as the driving force for the diffusion of liquid water.

In region 2, a noteworthy 38.4% of the total water loss diffused to the adjacent region in the form of liquid water during the preheating stage. This phenomenon can be attributed to a portion of the liquid water diffusing toward the surface of the coal slime dough under the influence of the pressure gradient. In contrast, region 3, which corresponds to the surface region, saw a substantial 76.7% of the total water loss migrating in the form of steam. This finding implies that the water evaporation across the entire slime mass predominantly occurred in the surface region.

During the constant temperature stage, there was a sharp increase in the proportion of water diffusing in the form of liquid water across all three regions. This surge was attributed to the establishment of a stable pressure gradient, driven by the pumping effect, both inside and outside the coal slime dough. This gradient effectively promoted the diffusion of internal liquid water toward the surface evaporation region. Particularly noteworthy was the fact that at 115 s of microwave action, a substantial 74.3% of the water loss in region 2 diffused to the surface of the coal slime as liquid water.

In the reheating stage, owing to the reduced water content within the coal slime, the formation of a liquid bridge between coal slime particles became challenging. Consequently, most of the water inside the coal slime dough diffused into the atmospheric environment in the form of water vapor.

#### The migration mechanism of moisture during microwave drying

**Fig. 5:**
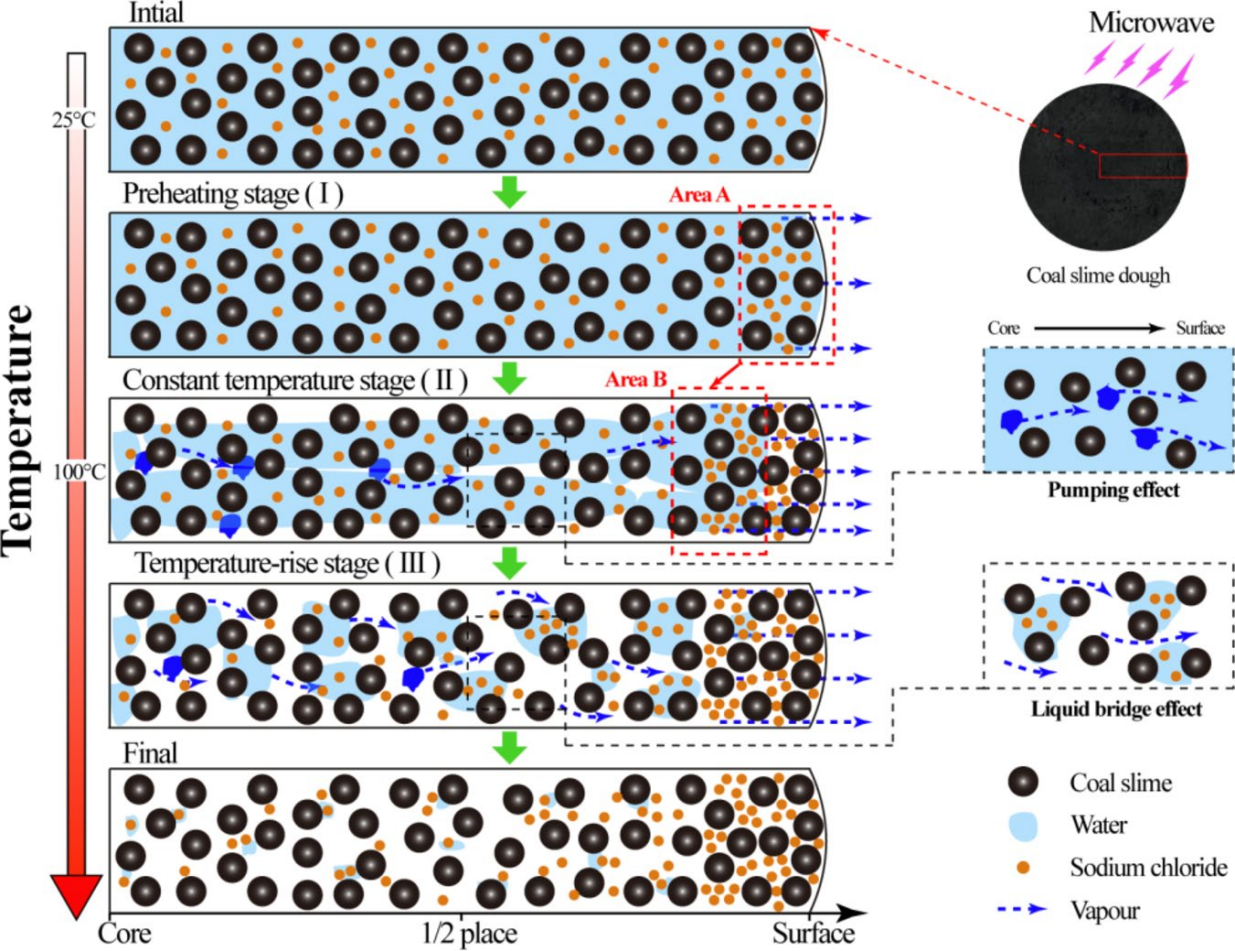
Schematic of moisture transmission and diffusion during microwave drying.

The moisture migration and diffusion mechanism during the microwave drying process of the coal slime dough can be elucidated based on the changing characteristics of moisture and chloride ion concentrations at different locations, as illustrated in Fig. 5.

Initial Stage: At the onset of microwave drying, liquid water was uniformly distributed within the coal slime dough. As microwave radiation commenced, the overall temperature of the coal slime increased, leading to the vaporization of some water in the inner regions into water vapor. This vaporization raised internal pressure. Driven by the resulting pressure gradient, liquid water migrated towards the surface of the coal slime, where it evaporated into the surrounding environment. This phenomenon is consistent with previous research results^34^.

Temperature Equilibrium Stage: When the overall temperature reached 100℃, a dynamic equilibrium was established between the energy absorbed by the coal slime under microwave radiation and the energy required for water evaporation within the coal slime. During this stage, the pressure gradient generated by the vapor continued to promote the ongoing diffusion of liquid water within the coal slime towards the surface. This process was facilitated by the pumping effect, after which the water evaporated on the coal slime surface and entered the environment. As microwave time increased, the evaporation site shifted from the surface layer (Area A) to the core (Area B).

Reheating Stage: As the moisture content decreased to a certain threshold, the energy absorbed by the coal slime under microwave radiation exceeded the energy required for water evaporation within the coal slime. This marked the entry into the reheating stage. In this stage, the amount of water within the coal slime was insufficient to establish a continuous liquid bridge^35^. Consequently, liquid water ceased to diffuse continuously to the exterior of the coal slime dough. Instead, it vaporized into a gaseous form and diffused from the gaps between coal slime particles into the surrounding environment^36^. With continued microwave radiation, moisture in the wet coal slime dough was continuously discharged in vapor form, culminating in the completion of the microwave drying process.

#### Verification of moisture diffusion mechanism in microwave drying coal slime dough through multi-physical field simulation

To validate the moisture diffusion mechanism in the microwave drying process, multi-physical field simulation was employed to elucidate the coupled characteristics of mass and heat transfer within the microwave field.

**Fig. 6:**
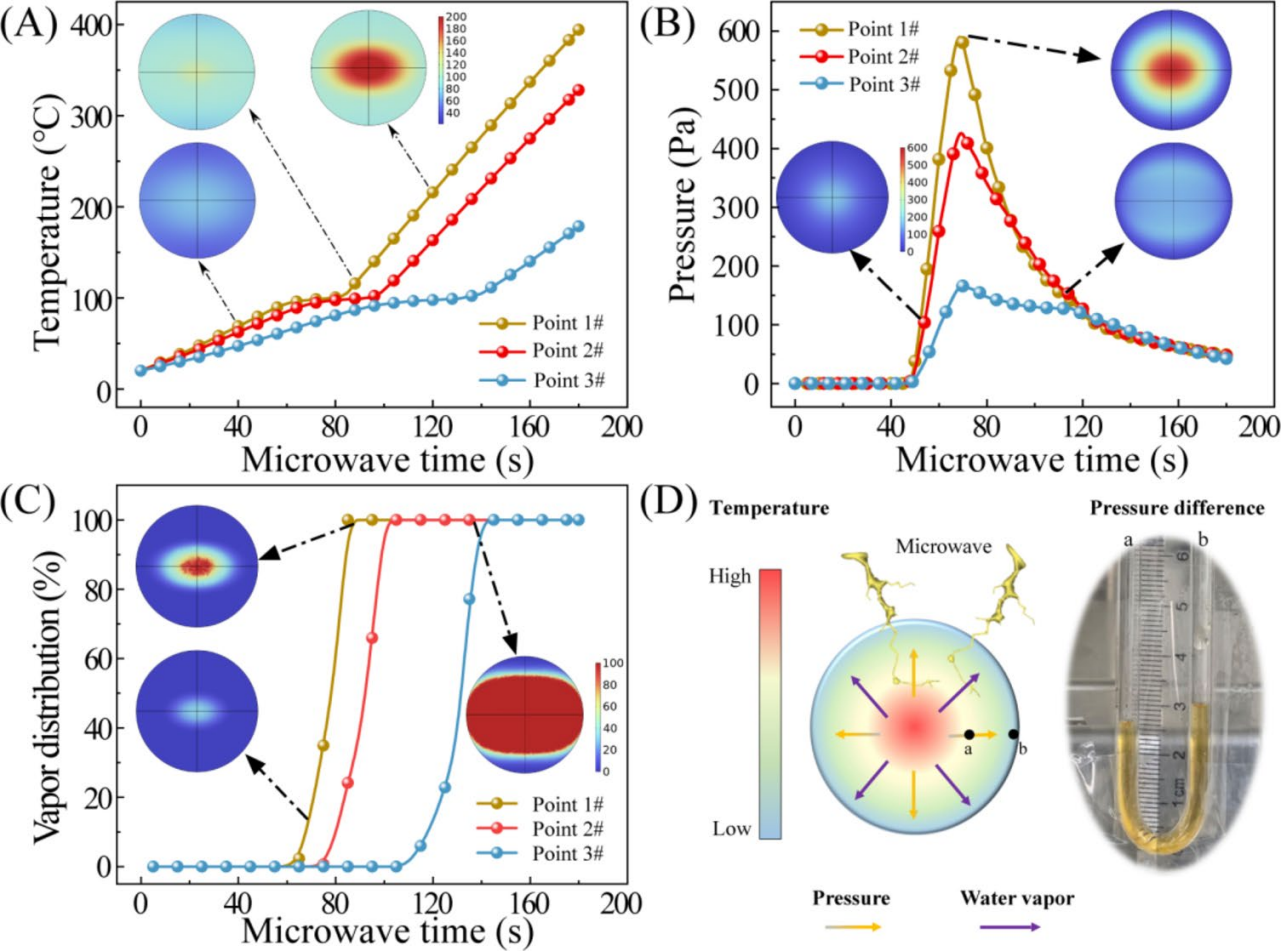
Distribution characteristics of temperature, pressure and water vapor in coal slime dough during microwave drying process.

The temperature profiles of the coal slime at three representative positions indicated that the temperature initially increased to 100 °C with the duration of microwave action and then gradually rose after a period of stability (as shown in Fig. 6A). Furthermore, the temperature cloud diagram of the coal slime dough cross-section revealed a distribution from high to low, extending from the center to the surface. This distribution pattern mirrored the temperature distribution observed in experimental measurements. As shown in Fig. 6B, the pressure within the coal slime at the three measurement points initially increased and then decreased with prolonged microwave exposure. This behavior was attributed to the vaporization of liquid water within the inner regions as the temperature increased, leading to a rise in the partial pressure of vapor inside the coal slime, as shown in Fig. 6C. The microscopic photo of water distribution in different regions was shown in figure S3. The pressure difference observed at different locations within the coal slime dough, as depicted in Fig. 6D, affirmed the accuracy of the simulation’s trends. In the later stages of drying, as the liquid water content decreased, the internal temperature of the coal slime increased once more, prompting gaseous water in the form of vapor to diffuse outward through the interstitial gaps between coal slime particles.

### Effect of moisture migration and diffusion

#### Influence of coal slime particle size

The size of coal slime particles plays a pivotal role in determining the pore structure and cross-sectional area of water diffusion channels within coal slime dough. Consequently, it significantly impacts the efficiency of water diffusion during microwave drying. To assess this effect, the microwave drying characteristics of coal slime with varying particle sizes, including 0.15–0.18 mm, 0.125–0.15 mm, 0.106–0.125 mm, and particles smaller than 0.075 mm, were investigated.

**Fig. 7:**
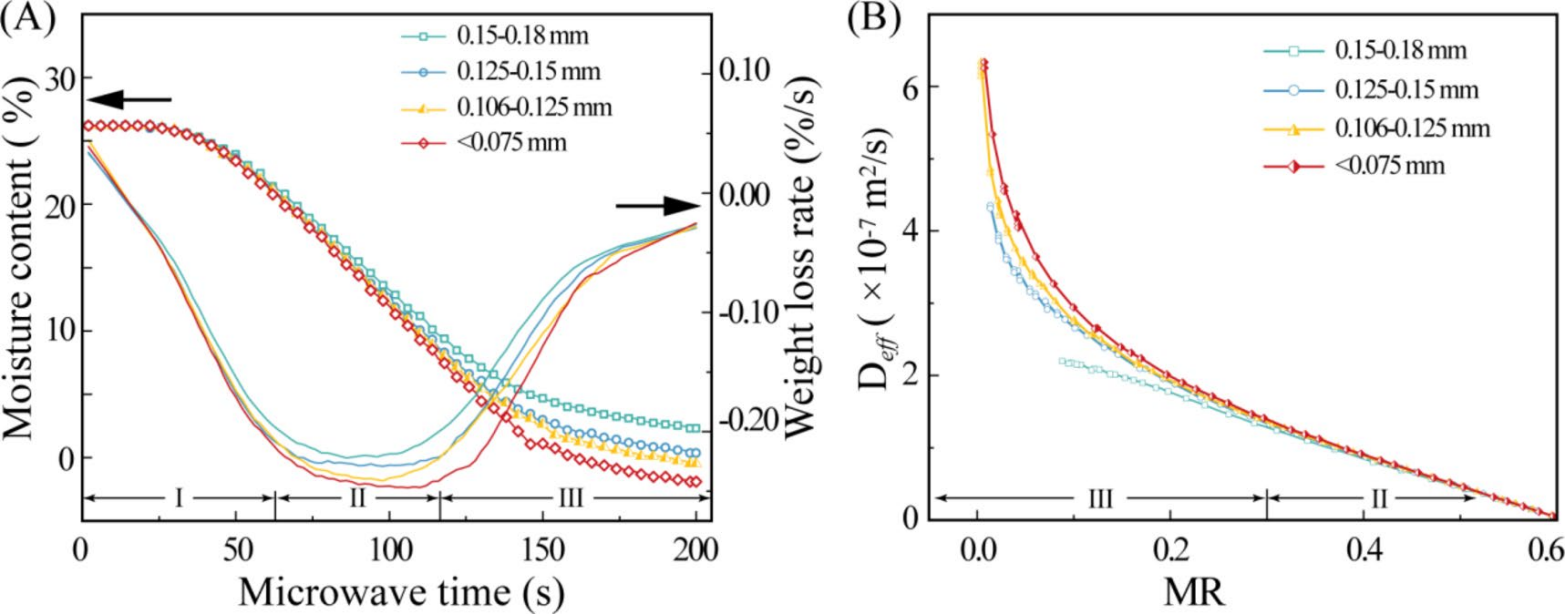
Variation law of moisture content and weight loss rate of coal slime with different particle sizes during microwave drying (**A**), variation curve of *D*_*eff*_ with MR (**B**). The microwave power was 300 W, and the diameter of the coal slime dough was 30 mm.

In Fig. 7A, it is evident that, within the same drying duration, coal slime with smaller particle sizes exhibited lower overall water content in the dough, particularly after the initial stage. This phenomenon can be attributed to the reduction in cross-sectional area of moisture diffusion channels formed between the smaller particles, resulting in enhanced “capillary effects” and improved drying efficiency^37^. In the later stages of the drying process, as water content decreased to a certain level, the capillary channels formed among smaller particles facilitated the formation of continuous liquid bridges by liquid water, thus promoting water migration towards the dough’s surface. Notably, with decreasing particle size, the maximum drying rate of spherical coal slime dough increased. This could be attributed to the fact that smaller coal slime particles provided a larger cross-sectional area for water diffusion channels between particles, facilitating easier internal-to-surface water diffusion. Additionally, smaller particle sizes resulted in larger evaporation areas for diffused water on the coal slime dough surface, leading to a stronger “pumping effect” induced by surface water evaporation^25^.

Fick’s law of diffusion-derived effective diffusion coefficients (D_*eff*_) have been widely employed to characterize the drying process^38^. To further investigate water diffusion characteristics during the later stages of drying, Fig. 7B illustrates the relationship between D_*eff*_ and moisture ratio (MR). As MR decreased, D_*eff*_ exhibited exponential growth. This behavior can be attributed to the rise in coal slime dough temperature after the preheating stage, leading to rapid surface moisture evaporation. This, in turn, created a water concentration gradient between the surface and the interior, promoting swift water diffusion from the interior to the exterior, driven by the “pumping effect.” Furthermore, it should be noted that smaller coal slime particle sizes were associated with larger effective diffusion coefficients in the later stages of drying. This suggests that liquid bridges formed more readily between smaller particles, facilitating liquid water diffusion to the exterior of the coal slime dough via capillary action.

#### Influence of coal slime dough diameter

The diameter of the material plays a significant role in determining the diffusion and transmission distance of moisture during the drying process. It is one of the key factors impacting the efficiency of the drying process. To elucidate the effects of coal slime dough diameter on drying characteristics, we investigated the microwave drying behaviors and moisture transportation in coal slime dough with diameters of 30 mm, 35 mm, and 40 mm.

**Fig. 8:**
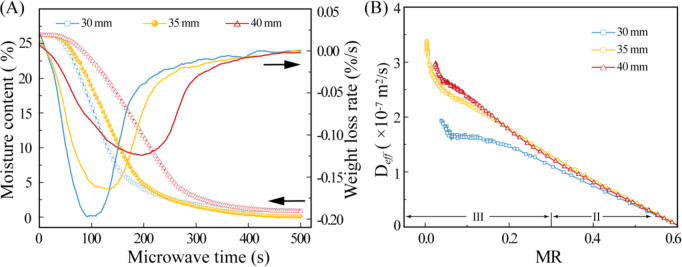
Variation law of moisture content and weight loss rate of coal slime with different diameters during microwave drying (**A**), variation curve of *D*_*eff*_ with *MR* (**B**). The microwave power was 300 W, and the particle size of slime particles was 0.15–0.18 mm.

Observing Fig. 8A, we note that as the diameter of the coal slime dough increases, the overall moisture content within the dough decreases within the same drying duration, particularly after the initial stage. This phenomenon arises from the increased moisture diffusion distance associated with larger diameters. As we progress into the second stage of the drying process, resistance along the diffusion channel increases, resulting in reduced microwave drying efficiency. Moreover, as the coal slime dough diameter increases from 30 mm to 40 mm, the time taken to reach the maximum drying rate increases (0.125%/s, 0.166%/s, and 0.197%/s, respectively).

This indicates that during the microwave drying process, the propagation of microwave energy within the coal slime diminishes progressively, resulting in reduced heating efficiency in larger coal slime agglomerates. Experimental results show that as the diameter of the coal slime agglomerates increases, the rate of internal moisture content change significantly decreases. This phenomenon suggests that the penetration depth of the microwaves is limited, and the insufficient microwave energy reaching the interior of larger agglomerates hinders the rapid migration and evaporation of internal moisture.

After the second stage, Fig. 8B displays the relationship between the effective diffusion coefficient (D_*eff*_) of coal slime dough with different diameters and the moisture ratio (MR). A decrease in MR corresponds to an increase in D_*eff*_, indicating that a significant portion of water in the drying process initially diffuses to the surface as liquid water due to the “pumping effect” and subsequently evaporates into water vapor. Furthermore, the effective diffusion coefficient increases during the later stages of drying, as larger coal slime dough diameters result in increased surface evaporation area and enhanced water concentration gradients between the interior and surface of the dough, amplifying the “pumping effect.”

## Conclusions

This study combines experimental research and multiphysics simulations to systematically analyze the mechanisms of moisture migration in coal slurry during microwave drying and quantitatively evaluates the moisture migration mechanisms at different stages. The main conclusions are as follows:

1. Based on the temperature distribution, the microwave drying process can be divided into three stages: preheating, constant temperature, and reheating.
2. During the preheating stage, moisture primarily evaporates from the surface, reducing the surface moisture content by 11.7%, while a small amount of internal liquid water transforms into vapor, promoting further moisture diffusion. In the constant temperature stage, moisture migrates to the surface via capillary action and vaporizes, with the evaporation point gradually shifting from the surface to the interior as surface moisture decreases. In the reheating stage, the remaining moisture mainly evaporates from within the coal slurry and diffuses into the surrounding environment.
3. During the drying process, smaller coal slurry particles exhibit a stronger capillary effect, leading to faster moisture migration and higher drying efficiency. In contrast, larger coal slurry particles, due to the longer diffusion distance for moisture, show lower drying efficiency.

This study provides an in-depth understanding of the moisture migration mechanisms during microwave drying, which is of significant importance for improving microwave drying technology, particularly for high-moisture and high-viscosity particulate materials. These findings contribute to expanding the knowledge of microwave drying processes for spherical materials and offer valuable insights for the design and optimization of drying technologies in various industrial applications.

## Electronic supplementary material

Below is the link to the electronic supplementary material.

Supplementary Material 1

## Data Availability

Data is provided within the supplementary information files.
