# Supplementary material for: Quantitative investigation of moisture migration during microwave drying of coal slime dough through simulation and tracer analysis
Source: Sci Rep. 2025 Apr 2;15:11308. doi: 10.1038/s41598-025-95646-y (PMC11965315; doi:10.1038/s41598-025-95646-y)
Supplement: Supplementary file 1 — Supplementary Material 1 [file 41598_2025_95646_MOESM1_ESM.docx]

**Supplementary Material**

Quantitative Investigation of Moisture Migration Mechanisms During Microwave Drying of Coal Slime Dough Through Multi-Physics Field Simulation and Tracer Analysis with NaCl

Fei Wang ^*^, Nan Tian, Lei Ren

*Engineering Research Center of Ministry of Education for Resource Efficiency Enhancing and Carbon Emission Reduction in Yellow River Basin Shanxi University, Taiyuan, Shanxi 030006, China;*

*Corresponding authors: Email: wangfei1859@sxu.edu.cn,

Full postal address: No. 92 Wucheng Road, Xiaodian District, Taiyuan City, Shanxi Province, China, 030006.


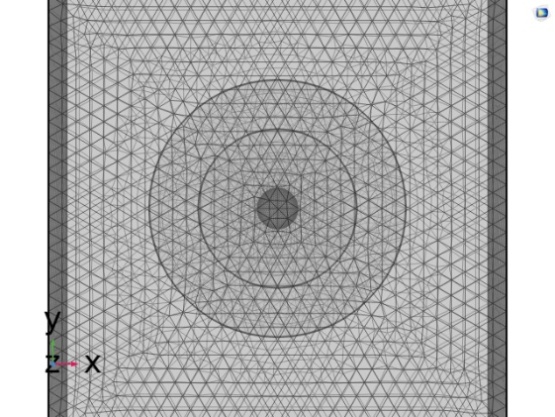


Figure S1. The mesh division of the geometric model

Table S1. Input parameters used in the simulations for microwave drying

| Content | Value | Unit |
| --- | --- | --- |
| Specific heat of dry coal slime | 1.6226 | KJ/(Kg·K) |
| Density of dry coal slime | 1500 | kg/m^3^ |
| Thermal conductivity | 0.46 | W/m/K |
| Initial moisture content of wet coal slime | 25.8 | % |
| Liquid water permeability | 10^-15^ | m^2^ |
| Gas phase inherent permeability | 1×10^−14^ | m^2^ |
| Porosity | 0.24 | % |
| Initial temperature | 20 | ℃ |
| Initial pressure | 1.0133 × 10^5^ | Pa |

Table S2 Dielectric properties of wet coal slime

| **Moisture content** | **0** | | **20%** | | **40%** | | **60%** | | **80%** | |
| --- | --- | --- | --- | --- | --- | --- | --- | --- | --- | --- |
| **Temperature/℃** | **Real part** | **Imaginary part** | **Real part** | **Imaginary part** | **Real part** | **Imaginary part** | **Real part** | **Imaginary part** | **Real part** | **Imaginary part** |
| 20 | 6.448 | 0.060 | 9.957 | 0.114 | 35.162 | 0.084 | 35.516 | 0.210 | 37.85 | 0.439 |
| 40 | 6.490 | 0.064 | 10.123 | 0.113 | 35.874 | 0.084 | 35.402 | 0.210 | 35.402 | 0.449 |
| 60 | 6.573 | 0.090 | 10.123 | 0.114 | 35.64 | 0.084 | 35.872 | 0.210 | 37.62 | 0.438 |
| 80 | 6.553 | 0.088 | 10.123 | 0.114 | 35.285 | 0.084 | 34.69 | 0.212 | 33.148 | 0.222 |
| 100 | 6.625 | 0.111 | 10.452 | 0.113 | 36.109 | 0.084 | 35.754 | 0.210 | 35.409 | 0.442 |
| 125 | 6.638 | 0.105 | 10.288 | 0.113 | 35.757 | 0.084 | 35.518 | 0.210 | 36.816 | 0.436 |
| 150 | 6.628 | 0.111 | 10.452 | 0.113 | 8.011 | 0.516 | 35.518 | 0.210 | 35.454 | 0.441 |
| 200 | 6.645 | 0.117 | 9.958 | 0.114 | 6.863 | 0.409 | 7.063 | 0.618 | 38.653 | 0.469 |

| 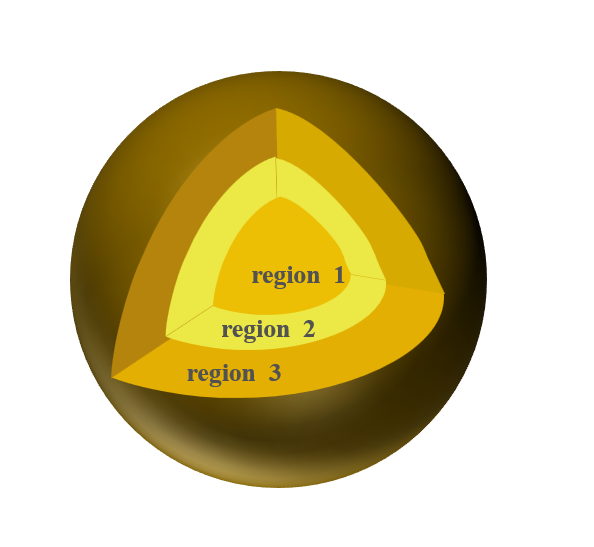 | 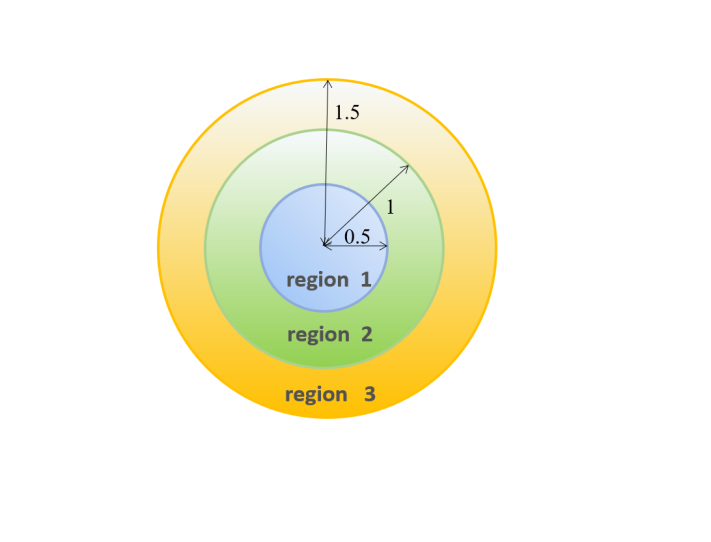 |
| --- | --- |
| Figure S2. Schematic diagram of coal slime dough zoning for calculating water migration in different regions | |


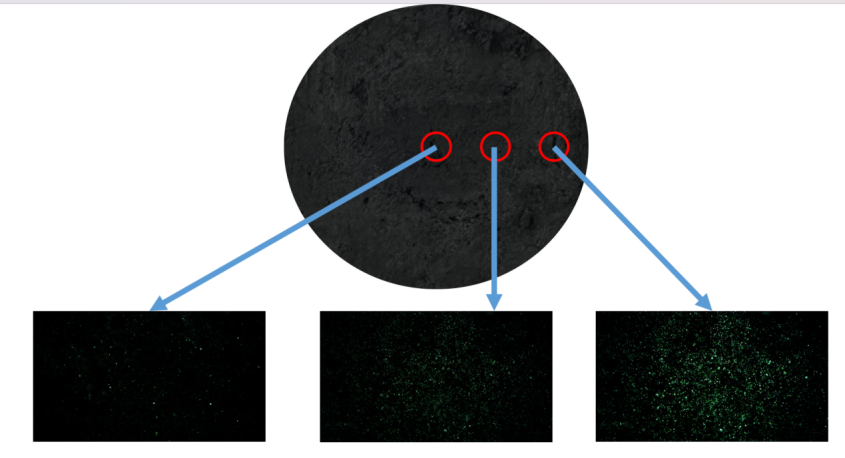


Fig. S3. Microscopic photos of water distribution in different regions at the reheating stage of drying (green dots represent liquid water)
